# A short-term longitudinal study of reciprocal relations among expectancy, task value, and achievement goals within the frameworks of the expectancy-value and achievement goal theories

**DOI:** 10.3389/fpsyg.2026.1720012

**Published:** 2026-04-10

**Authors:** Issei Manabe, Motoyuki Nakaya

**Affiliations:** Graduate School of Education and Human Development, Nagoya University, Nagoya, Aichi, Japan

**Keywords:** achievement goal theory, cross-lagged panel modeling, expectancy-value theory, learning motivation, longitudinal study, mastery goals, performance goals, task value

## Abstract

**Background:**

The expectancy-value and achievement-goal theories are two major frameworks for explaining individual differences in students’ learning motivation. Although both have accumulated substantial empirical support, their constructs are often examined in isolation, and little is known about their longitudinal and reciprocal relationships.

**Objective:**

This study investigated short-term longitudinal associations among expectancy, task value, cost, and achievement goals.

**Methods:**

A two-wave survey was conducted with 331 Japanese first-year university and vocational school students over 1 month. Participants completed validated questionnaires measuring expectancy, task value (interest, attainment, practical utility, and institutional utility), cost, and achievement goals (mastery, performance approach, and performance avoidance). Cross-lagged panel modeling was conducted using full information maximum likelihood estimation.

**Results:**

Institutional utility value at Time 1 positively predicted practical utility value at Time 2. Mastery goals at Time 1 positively predicted practical utility value, interest value, and attainment value at Time 2. Performance-avoidance goals at Time 1 positively predicted performance-approach goals at Time 2. In contrast, expectancy and task value showed limited predictive effects on subsequent achievement goals.

**Conclusion:**

These findings suggest that achievement goals, particularly mastery goals, may function as antecedents of specific expectancy-value components over time, whereas reverse pathways were not supported. The results highlight the importance of reconsidering the temporal ordering among motivational constructs and contribute to ongoing efforts to integrate Expectancy-Value Theory and Achievement Goal Theory.

## 1 Introduction

Understanding individual differences in learning motivation is a central concern in educational psychology. Various theoretical frameworks have been proposed to address these differences, among which the Expectancy-Value Theory and Achievement Goal Theory play pivotal roles in elucidating students’ motivation to learn (Elliot and Dweck, 2005; Wigfield and Eccles, 2000). Each theory focuses on the psychological factors that influence learning activities. However, in actual educational settings, multiple motivational factors likely interact in complex ways, influencing learner behavior and academic achievement. Consequently, a single theoretical framework may be insufficient to fully explain students’ motivation, highlighting the need for integrative investigations across theories (e.g., Schunk et al., 2008). Despite this, research combining these frameworks has been limited. Therefore, this study examines key constructs from both the Expectancy-Value Theory and Achievement Goal Theory.

Expectancy-Value Theory posits that learners’ performance and motivation on a given task are predicted by two factors: the extent to which they believe they are likely to succeed (expectancy) and the extent to which they perceive the task as valuable (task value). Expectancy refers to the perceived likelihood of success, whereas task value reflects the subjective importance or meaningfulness of the task to the learner.

Originally proposed by Atkinson (1957) and Atkinson and Feather (1966), the theory was primarily based on experimental procedures. Eccles et al. (1983) later expanded its application to educational contexts, and Eccles and Wigfield (2020) refined the framework through Situated Expectancy-Value Theory (SEVT). They emphasized that these constructs are situated within contexts and shaped by various developmental and social factors, including personal belief systems, self-schemas, and prior experiences of success and failure.

Task value is typically described as encompassing four dimensions: interest value, attainment value, utility value, and cost. Interest value reflects intrinsic enjoyment and curiosity toward the task; attainment value reflects the importance of task accomplishment for one’s beliefs or self-concept; utility value reflects the perceived usefulness of the task for future careers or everyday life; and cost reflects the negative aspects of tasks, such as effort, time, and psychological burden (e.g., Eccles and Wigfield, 2020). Additionally, Ida (2003) distinguished two types of utility value: practical utility value, referring to usefulness for professional practice, and institutional utility value, referring to usefulness for examinations and job hunting.

Kera and Nakaya (2014) revised practical utility value measures for junior high school students, arguing that adolescents find it difficult to imagine professional practice and that prior research often emphasized everyday relevance. Their revised items assessed how content could be applied in daily life. Their results indicated that practical utility value positively predicted interest in learning content, whereas institutional utility value positively predicted student engagement. These findings suggest that the two types of utility value influenced different learning-related outcomes, and this study distinguishes between them accordingly.

Empirical studies consistently support expectancy and task value as antecedents of academic motivation and performance. For example, Gaspard et al. (2015) demonstrated that enhancing task value in mathematics classes increased students’ motivation, while Trautwein et al. (2006) found that increasing students’ expectancies promoted greater homework effort. Many other studies report that expectancy and task value are positive predictors of learning outcomes (e.g., Hulleman et al., 2010; Nagengast et al., 2011).

Research has also examined reciprocal influences among Expectancy-Value Theory constructs. For instance, Perez et al. (2019) analyzed longitudinal data to investigate how expectancy and task value influence each other over time. Their findings suggest that expectancy positively influences attainment and interest values, attainment value negatively influences cost, and utility value positively influences attainment value. These results provide empirical evidence that Expectancy-Value Theory constructs are mutually influential.

In summary, empirical investigations of Expectancy-Value Theory constructs continue to accumulate, steadily advancing the theoretical foundation for predicting learning motivation and activities.

Achievement Goal Theory focuses on learners’ goal orientations when approaching tasks, such as the reasons for learning. Dweck (1986) and Nicholls (1984) proposed two categories of goals: mastery goals, which emphasize understanding and improving competence, and performance goals, which emphasize favorable evaluation by others. Building on this distinction, Elliot and McGregor (2001) introduced the approach–avoidance dimension, resulting in three types of goals: mastery (mastery-approach), performance-approach, and performance-avoidance.

Although a fourth category, mastery-avoidance goals, has been proposed—referring to avoiding failure while attempting to improve competence—it remains difficult to distinguish empirically from other goals and has rarely been studied (Elliot et al., 2011). Therefore, empirical studies in educational psychology typically measure goal orientation using the three-category frameworks of mastery-approach, performance-approach, and performance-avoidance goals.

Among these, mastery goals consistently associate with intrinsic motivation and persistence (e.g., Liem et al., 2008; Pintrich, 2000), suggesting positive influences on learning. In contrast, performance-avoidance goals are linked to test anxiety, lower academic performance, and avoidance behaviors (e.g., Anderman and Midgley, 1997; Midgley et al., 2001; Murayama and Elliot, 2009).

Achievement goals are also context-dependent and flexible. Models suggest that individuals may pursue multiple goals simultaneously (Barron and Harackiewicz, 2001), and goal selection is influenced by personal characteristics as well as contextual and educational factors (Senko et al., 2011).

Both Expectancy-Value Theory and Achievement Goal Theory have been developed to explain learning motivation, but they are grounded in different constructs and traditions. Expectancy-Value Theory emphasizes cognitive evaluations (“expectancies for success” and “subjective valuations of tasks”), whereas Achievement Goal Theory emphasizes goal orientations (“why learners engage in tasks”). Because these theories conceptualize motivation from complementary perspectives, integrating them could provide richer insights, yet they are often examined in isolation.

Several studies have attempted to examine constructs from both theories simultaneously. However, these studies have generally been limited to testing models with pre-assumed causal directions (e.g., Jiang et al., 2020; Plante et al., 2013) or parallel profiling approaches (e.g., Conley, 2012). For example, Conley (2012) conducted a typological analysis of motivational profiles integrating expectancy, task value, and achievement goals, but did not examine causal directions. Plante et al. (2013) tested models in which expectancy and task value influenced achievement directly and indirectly through achievement goals, but relied on cross-sectional data, leaving bidirectional relationships unexplored. Jiang et al. (2020) tested a prespecified causal structure, from task value to achievement goals, making it difficult to examine reciprocal relations.

In summary, although several empirical studies have investigated the constructs from both theories, few have used longitudinal designs to examine causal relationships or reciprocal relationships over time. Clarifying these associations can refine theoretical understanding, enhance prediction of learning behaviors and motivation (Plante et al., 2013) and inform educational interventions. Therefore, investigating longitudinal and reciprocal influences between constructs from both theories is a crucial task with theoretical and practical significance.

Building on this background, this study explores how key constructs of the Expectancy-Value Theory and Achievement Goal Theory are longitudinally related. In particular, it examines the possibility of reciprocal longitudinal influences using a cross-lagged panel model. This study aims to move beyond unidirectional models to clarify temporal dynamics. It should be noted that the study is exploratory; given that relationships among constructs of the two theories are not fully established, interactions between constructs are not examined here.

## 2 Methods

### 2.1 Participants and procedure

Participants were recruited using non-probabilistic sampling from two sources. First, first-year students enrolled in psychology-related courses at universities and vocational schools in Aichi Prefecture, Japan, were invited through course instructors. Second, first-year university and vocational students nationwide were recruited through an online research panel (Freeasy; iBridge Corporation). We focused on first-year students because the first year represents an early stage of postsecondary education during which students are adapting to a new academic environment and may form or recalibrate their expectancy-value beliefs and achievement goals.

The survey was administered online via Qualtrics at two time points: early December 2024 (T1) and early January 2025 (T2). Regular classes occurred during this interval, and no examinations were held. To ensure data quality, we embedded an attention-check item from the Directed Questions Scale (DQS; Maniaci and Rogge, 2014) at each wave. Responses were treated as valid for a given wave only when the participant provided informed consent and correctly answered the DQS item at that wave; participants who failed the DQS at both waves were excluded from the dataset.

After applying these criteria, the analytic dataset comprised 331 first-year students in total, including 149 with valid responses at both waves, 95 with valid responses only at T1, and 87 with valid responses only at T2. Because restricting analyses to complete cases can reduce statistical power and introduce selection bias, all primary analyses were conducted using full information maximum likelihood estimation to incorporate all available valid data.

This study was approved by the Research Ethics Committee of the authors’ graduate school (Approval No.: 24–2,329). All participants were informed of the study’s purpose and guaranteed anonymity before completing the survey.

### 2.2 Measures

All items were rated on a six-point Likert scale (1 = strongly disagree to 6 = strongly agree). Minor wording adjustments were made to each scale to suit the context of psychology courses for university students.

#### 2.2.1 Expectancy

Expectancy was measured using the Japanese version of the self-efficacy subscale of the Motivated Strategies for Learning Questionnaire (MSLQ; Pintrich et al., 1990; translated by Mori, 2004). This scale consists of nine items (e.g., “Compared to others, I think my learning ability is excellent”), all of which were used in this study.

#### 2.2.2 Task value

The task value was assessed using the Task Value Rating Scale developed by Kera and Nakaya (2014), with reference to Eccles and Wigfield (1985) and Ida (2001, 2003). The scale indicates four factors: practical utility value (three items; e.g., “I think the content of psychology is useful in my daily life”), institutional utility value (3 items; e.g., “Studying psychology will be useful when I look for a job”), interest value (4 items; e.g., “I find the content of psychology interesting”), and attainment value (3 items; e.g., “Understanding the content of psychology helps me grow as a person”). All 13 items across the four factors were used in this study.

#### 2.2.3 Cost

Cost was measured using the short form of the Cost Scale developed by Beymer et al. (2022), which includes four items drawn from one of the four factors of the original Cost Scale by Flake et al. (2015). The items were translated into Japanese and back-translated to ensure accuracy.

#### 2.2.4 Achievement goals

Achievement goals were assessed using the Goal Orientation Scale developed by Okada et al. (2012) based on Elliot and Church (1997) and Tanaka and Yamauchi (2000). This scale measures three types of goals: mastery goals (5 items; e.g., “In psychology classes, I think it is important to study new things even if they are somewhat difficult”), performance-approach goals (5 items; e.g., “In psychology classes, I aim to achieve better grades than others”), and performance-avoidance goals (5 items; e.g., “In psychology classes, I do not want others to think I am not smart because I get poor test scores”). All 15 items across the three factors were included in this study.

### 2.3 Data analysis

Descriptive statistics and correlation coefficients were calculated using pairwise deletion. Internal consistency was evaluated using Cronbach’s *α* and McDonald’s *ω* for each scale at T1 and T2.

Because the cross-lagged panel model was estimated using composite scores rather than latent variables, longitudinal measurement invariance was examined for each construct prior to the main analysis. Confirmatory factor analyses were conducted separately for T1 and T2 indicators, and configural, metric, and scalar invariance models were tested by imposing equality constraints on factor loadings and, subsequently, item intercepts across time. Model comparisons were evaluated using changes in fit indices, with ΔCFI ≤ 0.010 and ΔRMSEA ≤ 0.015 as criteria for invariance.

The cross-lagged panel model was then estimated using structural equation modeling. Missing data were handled using full information maximum likelihood estimation. To control for inflation of Type I error due to multiple cross-lagged paths, *p*-values associated with cross-lagged regression coefficients were adjusted using the Benjamini–Hochberg procedure. Model fit was evaluated using the CFI, TLI, RMSEA, and SRMR.

## 3 Results

### 3.1 Descriptive statistics, internal consistency, and attrition analyses

Table 1 presents the descriptive statistics and reliability coefficients for each scale at T1 and T2. Cronbach’s α and McDonald’s ω coefficients ranged from 0.73 to 0.93 across all scales, indicating sufficient internal consistency. The correlations among variables are presented in the Supplementary material.

**Table 1:** Descriptive statistics.

Variables		T1					T2				
		*N*	*M*	*SD*	*α*	*ω*	*N*	*M*	*SD*	*α*	*ω*
1.	Expectancy	233	3.44	0.87	0.90	0.90	240	3.51	0.92	0.93	0.93
2.	Practical utility value	236	4.20	0.98	0.82	0.82	244	4.23	0.95	0.82	0.82
3.	Institutional utility value	236	4.00	1.04	0.82	0.82	243	4.07	1.00	0.81	0.82
4.	Interest value	236	3.98	0.72	0.83	0.85	244	4.00	0.68	0.82	0.84
5.	Attainment value	235	4.17	0.96	0.74	0.75	244	4.25	0.92	0.73	0.73
6.	Cost	236	2.44	1.08	0.84	0.84	244	2.42	1.03	0.83	0.84
7.	Mastery goal	235	4.13	0.88	0.84	0.84	240	4.20	0.87	0.85	0.85
8.	Performance approach goal	236	3.48	1.06	0.86	0.87	242	3.58	1.10	0.89	0.89
9.	Performance avoidance goal	234	3.71	1.14	0.88	0.88	243	3.61	1.29	0.91	0.91

To examine potential attrition bias, participants who completed both waves were compared with those who responded only at T1 on baseline variables. Independent samples t-tests indicated that participants who did not complete T2 reported significantly higher cost at T1 than those who completed both waves, with a medium effect size of approximately d = 0.62. No significant differences were observed for the other motivational variables.

These results suggest that participants with higher perceived cost at T1 were more likely to discontinue participation at T2. Although most baseline motivational variables did not differ between groups, the observed difference in cost indicates that attrition was not entirely random. This pattern is taken into account when interpreting the longitudinal findings.

### 3.2 Longitudinal measurement invariance

Before estimating the cross-lagged panel model using composite scores, longitudinal measurement invariance was examined for each construct across T1 and T2 using confirmatory factor analysis (Table 2).

**Table 2:** Path coefficients from T1 to T2 for the same variables.

Variable		*b*	*SE*	*β*
1.	Expectancy	0.674	0.047	0.662
2.	Practical utility value	0.583	0.046	0.638
3.	Institutional utility value	0.613	0.048	0.654
4.	Interest value	0.350	0.060	0.380
5.	Attainment value	0.281	0.066	0.304
6.	Cost	0.802	0.047	0.811
7.	Mastery goal	0.657	0.047	0.683
8.	Performance approach goal	0.495	0.056	0.495
9.	Performance avoidance goal	0.785	0.052	0.739

All path coefficients were significant at *p* < 0.001.

For each construct, three nested models were tested sequentially: configural invariance, metric invariance with factor loadings constrained equal across time, and scalar invariance with both loadings and item intercepts constrained equal. Model comparisons were evaluated using changes in fit indices, with *Δ*CFI ≤ 0.010 and *Δ*RMSEA ≤ 0.015 indicating acceptable invariance.

Across all constructs, changes in model fit between configural, metric, and scalar models were within the recommended thresholds. These results support at least scalar invariance across time for all constructs, indicating that the relationships between observed indicators and their underlying latent variables remained stable across measurement occasions. Therefore, the use of composite scores in the subsequent cross-lagged analyses was considered psychometrically justified.

### 3.3 Cross-lagged panel model

To examine the longitudinal relationships among expectancy, task value, and achievement goals across the two time points, a cross-lagged panel model was constructed and analyzed using structural equation modeling. The model included covariances among all variables at T1 and the error terms of all variables at T2. Paths with *p*-values ≥ 0.05 or standardized path coefficients below 0.10 were sequentially removed, and the analysis was repeated until only significant paths remained. The final model demonstrated an acceptable fit (CFI = 0.987, TLI = 0.970, RMSEA = 0.041, and SRMR = 0.061). After adjusting for multiple comparisons using the Benjamini–Hochberg procedure, several cross-lagged effects remained statistically significant.

All the autoregressive paths from T1 to the same construct at T2 were significant, confirming the temporal stability of each variable (2).

**Figure 1:**
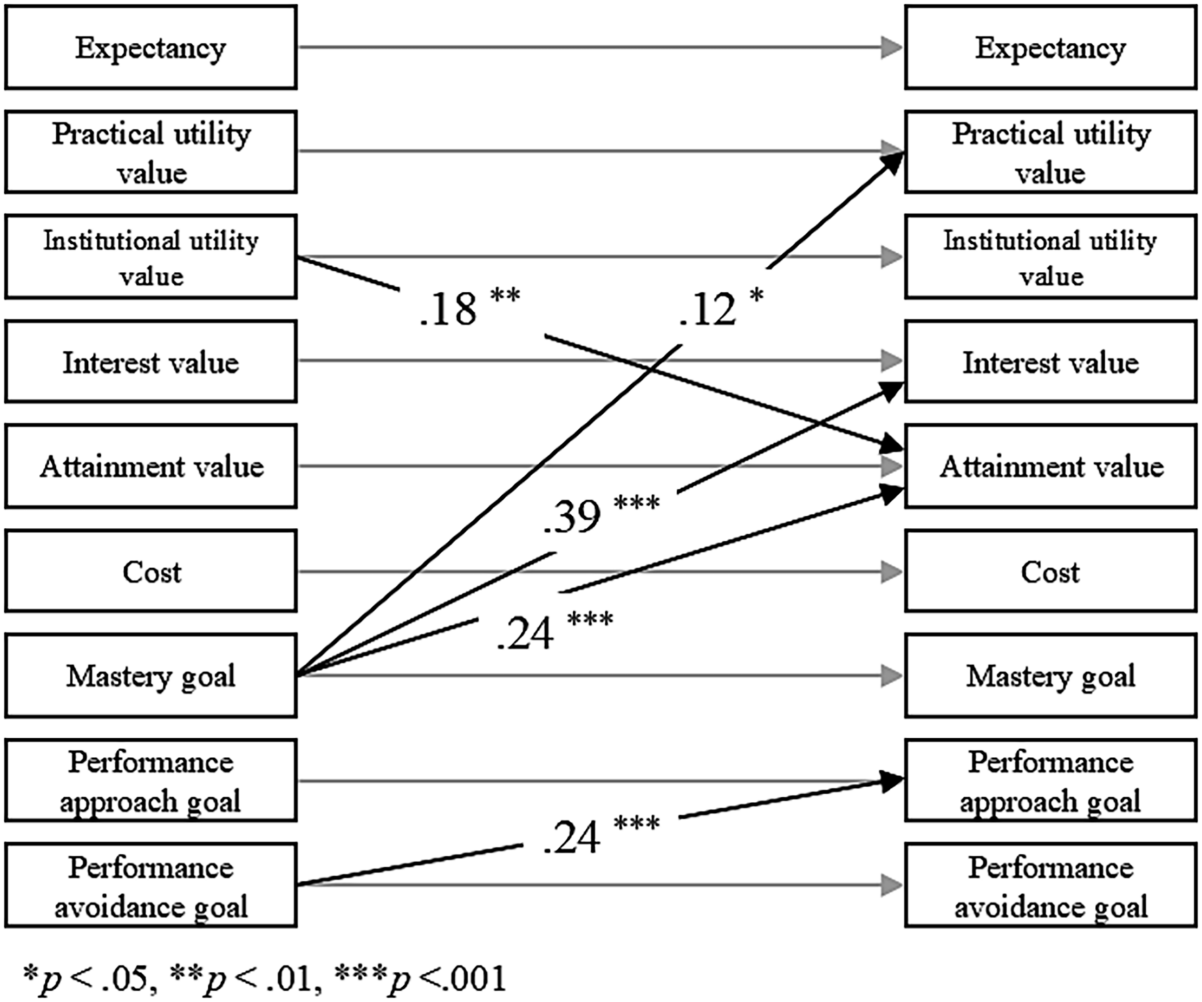
Cross-lagged panel model of expectancy, task values, and achievement goals from T1 to T2.

Significant cross-lagged paths from T1 to different constructs at T2 are as summarized in Figure 1.

Institutional utility value at T1 positively predicted practical utility value at T2 (b = 0.151, SE = 0.057, *β* = 0.176, *p* < 0.01). Mastery goals at T1 positively predicted practical utility value (b = 0.120, SE = 0.060, *β* = 0.116, *p* < 0.05), interest value (b = 0.299, SE = 0.051, *β* = 0.393, *p* < 0.001), and attainment value (b = 0.247, SE = 0.065, *β* = 0.243, *p* < 0.001) at T2. Performance-avoidance goals at T1 positively predicted performance-approach goals at T2 (b = 0.230, SE = 0.058, *β* = 0.244, *p* < 0.001).

Overall, the findings suggest that achievement goals may function as antecedents of specific expectancy–value components over time, whereas reverse pathways were not supported.

## 4 Discussion

### 4.1 Reciprocal relations among expectancy, task value, and achievement goals

This study examined the longitudinal influences of key constructs from the Expectancy-Value Theory and Achievement Goal Theory using a cross-lagged panel model. The analyses revealed that mastery goals exerted positive effects on subsequent; practical utility value, interest value, and attainment value. In contrast, the effects of expectancy and task value on achievement goals were limited. These findings suggest that mastery goals may function as antecedents of specific task value components, offering an alternative perspective on the directional relations proposed in prior research.

### 4.2 Influence of mastery goals on task value

The finding that mastery goals positively influence subsequent task values contrasts with prior research (Plante et al., 2013; Jiang et al., 2020), which assumed that expectancies and values drive achievement goals. Rather, the present results suggest a directional longitudinal pattern in which mastery goals precede certain components of task value over time.

One possible explanation is that engaging in learning with mastery goals fosters active involvement, thereby enhancing task value. For instance, students who approach psychology classes with mastery goals are more likely to deepen their understanding of the content and explore related concepts. Such engagement may consolidate knowledge and strengthen recognition of the content’s usefulness and personal importance.

Another possible explanation concerns differences in situatedness between the constructs derived from Expectancy-Value Theory and Achievement Goal Theory. Expectancy-value components are often conceptualized as relatively context-sensitive and responsive to immediate learning experiences, whereas goal orientations have historically been described as broader cognitive-motivational frameworks that shape how students interpret and approach academic tasks (Maehr and Zusho, 2009). If mastery goals operate at a more generalized level of self-organization, they may function as relatively enduring self-schemata that guide the development of more situation-specific value beliefs over time. From this perspective, the stronger predictive role of mastery goals across a one-month interval may reflect differences in the level of abstraction and temporal stability at which these motivational constructs operate.

Examining these pathways provides a flexible perspective on the causal relations among motivational constructs. Future longitudinal and empirical studies are needed to clarify the dynamics of these relationships.

Prior research has explored ways to promote mastery goals. The TARGET framework (Ames, 1992) emphasizes structuring the learning environment to foster mastery goals, while more recent research highlights the role of autonomy-supportive teaching (Benita and Matos, 2021). Together with the present findings, this evidence underscores the value of interventions that strengthen mastery goals as one potential entry point for supporting the development of value beliefs.

Elliot and Sommet (2023) have called for an integrative understanding of motivational constructs through the goal complex framework, which combines energization (i.e., task value) as the driver of behavior with direction (i.e., achievement goals) as the guide. While the goal complex framework allows for reciprocal influences between energization and direction, the present study primarily supports a directional pathway from direction to energization, in that mastery goals predicted subsequent task value components. Future research should adopt this perspective and examine reciprocal interactions and temporal dynamics using extended longitudinal and empirical studies.

### 4.3 Limitations and future directions

This study had several limitations. First, the survey was conducted at only two time points, T1 and T2, 1 month apart. Longer-term multiwave designs are needed to capture the dynamics of motivational change processes more precisely. Second, the sample was limited to university and vocational students in Japan, raising the possibility that cultural and educational contexts influenced the results. Cross-cultural validation is therefore necessary. Third, the cross-lagged panel model was specified using composite scores rather than latent variables. Although longitudinal measurement invariance was supported, future research should examine whether similar patterns are obtained using latent variable approaches when feasible. Fourth, motivation is influenced not only by learners’ internal cognition but also by contextual factors such as teachers’ instructional styles and classroom climate (e.g., Senko et al., 2011). These external influences warrant consideration in future research.

In light of these limitations, future research should explore the bidirectional relationships among expectancy, task value, and achievement goals, and evaluate the effectiveness of educational practices designed to foster adaptive motivation and learning behaviors.

## 5 Conclusion

This study examined the interrelations among key constructs of Expectancy-Value Theory and Achievement Goal Theory using short-term longitudinal data. The results demonstrated that mastery goals exerted positive effects on specific subsequent task value components, suggesting a directional longitudinal pattern that differs from the traditional assumption that task value primarily precedes achievement goals. These findings highlight the importance of reconsidering the temporal ordering among motivational constructs and offer implications for both theoretical refinement and educational practice.

## Data Availability

The raw data supporting the conclusions of this article will be made available by the authors, without undue reservation.
