# Supplementary material for: A short-term longitudinal study of reciprocal relations among expectancy, task value, and achievement goals within the frameworks of the expectancy-value and achievement goal theories
Source: Front Psychol. 2026 Apr 10;17:1720012. doi: 10.3389/fpsyg.2026.1720012 (PMC13106477; doi:10.3389/fpsyg.2026.1720012)
Supplement: Supplementary file 1 [file Data_Sheet_1.PDF]

Supplementary Material. Correlations among variables

Variables	1	2	3	4	5	6	7	8	9	10	11	12	13	14	15	16	17
1. Expectancy T1	—																
2. PU value T1	.39***	—															
3. IU value T1	.32***	.70***	—														
4. Interest value T1	.40***	.76***	.66***	—													
5. Attainment value T1	.37***	.80***	.73***	.70***	—												
6. Cost T1	-.23**	-.57***	-.41***	-.56***	-.47***	—											
7. Mastery goal T1	.40***	.75***	.57***	.75***	.69***	-.58***	—										
8. PApG T1	.39***	.25**	.25**	.21*	.26*	-.10	.37***	—									
9. PAvG T1	.14	.22**	.15	.16	.26*	-.06	.32***	.74***	—								
10. Expectancy T2	.68***	.36***	.38***	.37***	.37***	-.19*	.43***	.34***	.14	—							
11. PU value T2	.30***	.82***	.63***	.67***	.70***	-.49***	.71***	.14	.15	.48***	—						
12. IU value T2	.24**	.69***	.77***	.56***	.66***	-.45***	.58***	.14	.12	.42***	.78***	—					
13. Interest T2	.32***	.72***	.61***	.75***	.63***	-.70***	.78***	.14	.10	.48***	.76***	.70***	—				
14. Attainment value T2	.26**	.71***	.63***	.62***	.72***	-.46***	.71***	.22**	.25**	.46***	.83***	.76***	.76***	—			
15. Cost T2	-.25**	-.45***	-.37***	-.47***	-.39***	.85***	-.49***	-.04	.02	-.20*	-.39***	-.45***	-.60***	-.35***	—		
16. Mastery goal T2	.41***	.64***	.52***	.61***	.63***	-.49***	.81***	.30***	.25**	.63***	.79***	.63***	.78***	.76***	-.44***	—	
17. PApG T2	.34***	.27***	.20*	.15	.31***	-.05	.36***	.72***	.63***	.47***	.27***	.21**	.18*	.34***	-.01	.45***	—
18. PAvG T2	.13	.19*	.05	.09	.20*	.01	.28***	.62***	.77***	.23**	.18*	.08	.07	.24**	.08	.30***	.80***

PU value = Practical Utility value, IU value = Institutional Utility value, PApG = Performance Approach Goal, PAvG = Performance Avoidance Goal

*$p < .05$, **$p < .01$, ***$p < .001$
